# Psychosocial-spiritual well-being is related to resilience and mindfulness in patients with severe and/or life-limiting medical illness

**DOI:** 10.1186/s12904-023-01258-6

**Published:** 2023-09-11

**Authors:** Polycarpe Bagereka, Rezvan Ameli, Ninet Sinaii, Marcelli Cristine Vocci, Ann Berger

**Affiliations:** 1 Pain and Palliative Care Service, Clinical Center, National Institutes of Health, Bethesda, MD USA; 2 Biostatistics and Clinical Epidemiology Service, Clinical Center, National Institutes of Health, Bethesda, MD USA

**Keywords:** Psychosocial-spiritual well-being, Resilience, Mindfulness

## Abstract

**Background:**

Improvement of psychosocial-spiritual well-being in patients with life-threatening or life-limiting illness is desirable. Resilience and mindfulness are considered to be helpful for enhancing psychosocial-spiritual well-being. Mindfulness-based interventions have been shown to promote resilience to stress and enhance well-being. However, in medical patients, evidence for the associations between mindfulness and resilience is lacking. We hypothesize patients with higher levels of psychosocial-spiritual well-being demonstrate greater resilience and mindfulness.

**Methods:**

200 patients (mean age = 50.2, SD = 15.5) with serious and or life-limiting illnesses were recruited from the NIH Clinical Center. Patients completed a demographic questionnaire, the NIH-HEALS measure of psychosocial-spiritual well-being, the Connor-Davidson Resilience Scale (CD-RISC-10), and the Mindful Attention Awareness Scale (MAAS). The demographic questionnaire also included a question on current stress level.

**Results:**

The NIH-HEALS was positively correlated to CD-RISC-10 (r_s_=0.44, p < 0.001) and MAAS (r_s_=0.32, p < 0.001). These findings were consistent across all three NIH-HEALS factors. Additionally, CD-RISC-10 and MAAS demonstrated a meaningful relationship to each other (r_s_=0.46, p < 0.001). All three constructs were inversely related to current stress level.

**Conclusions:**

Findings suggest that there is a meaningful relationship between psychosocial-spiritual well-being, mindfulness, and resilience. Mindfulness and resilience are positively correlated in a medical population. Clinical interventions aimed at enhancing psychosocial-spiritual well-being through mindfulness and resilience can be highly promising for patients with severe and or life limiting illness.

## Introduction

Psychosocial-spiritual well-being is a multifaceted concept including psychological, social, and spiritual determinants of health [1]. It is correlated with general coping ability, lower stress levels, and better quality of life in patients with life-threatening illness, including advanced cancer [2, 3], cardiovascular diseases [4–6], and immune-compromised illnesses [7]. Psychosocial-spiritual well-being is closely related to the concept of total pain, developed by hospice pioneer Cicely Saunders [8], to describe the suffering experienced by patients battling terminal illness. Suffering, per se, is not simply physical pain, but a complex interaction between physical, emotional, social, and spiritual distress [2, 9]. Accordingly, reducing suffering requires treatment plans that address not only somatic but also psychosocial and spiritual needs of patients. As such, psychosocial-spiritual well-being is an important aspect of healing assessed by palliative care and other physicians to address clinical needs of patients [10, 11].

Psychosocial-spiritual well-being outcomes can be enhanced by resilience and mindfulness [12]. Resilience has been defined as successfully adapting to challenging life stressors and experiences [13, 14]. Resilience is considered to be a dynamic process that changes over time and across circumstances or stressors [15–17]. Mindfulness, defined as paying attention on purpose and in the present moment and nonjudgmentally [18], is known to promote health and well-being at physical, psychological, and spiritual levels in various clinical groups [19–22]. Resilience and mindfulness may be considered as related constructs enhancing psychosocial-spiritual well-being. Recent works with non-medical populations show that resilience mediates the relationship between mindfulness and several psychological outcomes and subjective well-being factors including life satisfaction, reduction of depression, and other negative affective experiences [23, 24].

Resilience has been shown to improve quality of life in patient with hypertension [25], glaucoma [26], depression [27], and cancer [28–30]. Protective factors or personal characteristics such as positive attitude, meaning, and social support may enhance resilience and help to successfully adapt or cope with illness [16, 31]. Resilience has also been associated with post traumatic growth which are positive life changes resulting from major life crises or stressful events [16]. Dealing with adversity can result in stronger relationships with friends and family and greater appreciation for life, which can increase the individual’s resilience and personal strength against life’s challenges [32].

Mindfulness has been identified as an essential protective factor that promotes resilience against psychological distress [33, 34]. Mindfulness relieves symptoms of depression, anxiety, and addiction in several clinical populations [35, 36]. Mindfulness enhances resilience through its decentering process or the ability to observe negative thoughts and emotions without judgment. Decentering can lead to increased distress tolerance, reduced emotional reactivity, and decreased over-engagement with negative thoughts and emotions [34, 37]. Thus, mindfulness-based interventions have been shown to promote psychological resilience to stress and enhance well-being in the general population and several healthcare and academic settings including among nurses, clinicians, and university students [21, 33, 38, 39].

Interestingly, the relationship between mindfulness and resilience has not been well-studied in clinical populations [39]. To our knowledge, this is the first study that reports the relationship between these two constructs in a relatively large cohort of severely ill patients. A strong correlation between mindfulness and psychosocial-spiritual well-being would indicate that mindfulness-based interventions could be promising in promoting resilience hence providing beneficial clinical and psychological outcomes in patient populations.

The present study investigates the relationship between psychosocial-spiritual well-being, with two constructs of resilience and mindfulness in patients with severe and or life-limiting illnesses. We predict (1) a strong relationship between psychosocial-spiritual well-being and resilience; (2) a strong relationship between psychosocial-spiritual well-being and mindfulness; and (3) a strong association between resilience and mindfulness. We further hypothesize that these constructs will be related to current stress level in patients.

## Methods

### Study design

The present study is part of a larger study that validated the National Institutes of Health (NIH) Healing Experiences of All Life Stressors (NIH-HEALS), a measure of psychosocial-spiritual healing developed by the Pain and Palliative Care Service (PPCS) at the NIH Clinical Center (CC). Additional information about the study design is detailed elsewhere [40].The study was approved by the NIH Office of Human Subject Research Protection (OHSRP). OHSRP deemed the current study Institutional Review Board (IRB) exempt as data were collected deidentified and study procedures only included paper-pencil questionnaires and were noninvasive. The requirement for signed written consent was waived by OHSRP, however, verbal consent was obtained from each participant prior to study procedures.

### Participants

Two hundred (200) patients from the NIH Clinical Center (CC) participated in the study, aged 18–89 years. All patients included in the study who were recruited from both the inpatient and outpatient clinics at the NIH CC, had serious and/or life-limiting medical illnesses and were already participating in experimental treatments and research projects related to their specific diseases. The majority of patients had advanced/metastatic cancer in one or several organs, while other conditions such as blood dyscrasias, graft vs. host disease (GVHD), and rare genetic conditions were also present (Table 1). Table 1 summarizes patient demographics.

### Procedure

Patients at various hospital units and Outpatient Clinics of CC were approached by a representative of the PPCS while they were in their hospital rooms or waiting for their medical appointments in outpatient settings. The PPCS representative described the study, and following verbal consent, participants were administered a packet of questionnaires to complete within the same day. The questionnaire packet contained documents that detailed the study, its objectives, the voluntary nature of participation, the type of information sought, the estimated time required to complete the questionnaires, and a clear explanation that declining to participate would not impact the patient’s care at the NIH.

### Measures

The study measures included the NIH-HEALS [40], the Connor-Davidson Resilience Scale (CD-RISC 10 item version) [41], the Mindful Attention Awareness Scale (MAAS) [42], and a study questionnaire with a question pertaining to current stress level. The NIH-HEALS has been validated by Ameli et al. [40].

NIH-HEALS is a 35-item self-report measure of psychosocial-spiritual well-being [40]. It is scored on a five-point Likert scale from Strongly Disagree [1] to Strongly Agree [5]. Four items have reversed scoring (items 6, 23, 28, and 34). NIH-HEALS has an internal consistency of 0.89 (Cronbach’s α > 0.7 is reliable) and includes three factors, namely Connection (10 items), Reflection & Introspection (14 items), and Trust & Acceptance (11 items). Its divergent and convergent validity have also been well-established [40]. While there is no difference between genders in the NIH-HEALS total score, women have been reported to score higher on items 5, 10, 31, and 35 of the Reflection & Introspection factor [43].

CD-RISC-10 is a shortened version of the 25-item CD-RISC scale by Connor and Davidson [44]. CD-RISC-10 is a 10 item self-report scale scored on a five-point Likert scale rated from 0 (Not true at all) to 4 (True nearly all the time). CD-RISC-10 measures resilience and the ability to cope with adversity [41]. The scale has demonstrated good construct validity and internal consistency in various populations (α = 0.85). Higher scores indicate higher levels of resilience.

MAAS is a 15 item self-report scale that assesses present moment awareness or mindfulness [42]. MAAS is scored on a six-point Likert scale rated from 1 (Almost Always) to 6 (Almost Never). Higher scores indicate higher levels of mindfulness. MAAS has demonstrated strong internal consistency with Cronbach’s α between 0.82 and 0.87 [42].

The study questionnaire also included a question on current level of stress that was assessed on an ordinal scale from “No stress” to “Extreme stress.”

**Table 1:** Demographics and raw scores*

	N(%)	
Gender		
Female	103(53)	
Male	90(43)	
Race & ethnicity		
Caucasian	139(72)	
Black or African American	30(16)	
Asian	13(7)	
Other	10(6)	
Hispanic or Latinx	13(7)	
Medical Diagnosis		
Cancer (advanced/metastatic)	128(70)	
Severe and/or Rare Non-Genetic conditions	20(11)	
Blood Dyscrasias	17(9)	
Severe and/or Rare Genetic conditions	16(9)	
AIDS	1(1)	
	**Median (IQR)**	**Range**
NIH-HEALS		
Total score	134 (120–145)	87–172
Connection factor score	40 (31–46)	13–50
Reflection & Introspection score	54 (50–60)	38–70
Trust & Acceptance score	41 (36–45)	20–55
CD-RISC-10		
Total score	31 (25–35)	10–40
MAAS		
Total score	67 (57–75)	15–90

*Adapted from Table 1. *Subject demographics of enrolled participants with serious and life-threatening disease*, Ameli et al. 2018 [40].

### Statistics

Data are described by frequencies and percentages, and simple descriptive statistics, i.e. mean ± standard deviation (SD) or median [inter-quartile (25th -75th percentile) range, IQR]. Data were assessed for the assumption of normality, and appropriate methods were used as indicated. Continuous data between two groups were compared using Wilcoxon rank sum tests, and among three or more categories using mixed models. Post-hoc pairwise comparisons were corrected for multiple comparisons using the Bonferroni method, and corrected p-values are reported. Spearman’s rho statistics were used for assessing correlations between measures. Results were interpreted based on the magnitude of effects (i.e., strength of the correlation) along with p-values. Data were analyzed using SAS v9.4 (SAS Institute, Inc, Cary, NC).

## Results

The sample included 200 patients with an age range of 18–89 years and mean of 50.2 ± 15.5 years. Of the 193 participants who indicated their gender, 53% identified as women and 47% identified as men. No other gender identification was made. Patients were Caucasian (72%), Black or African American (16%), or Asian (7%). Of the whole sample, 7% identified their ethnicity as Hispanic or Latinx. Subjects reported their religious affiliations as Christian (66%), Not Affiliated (12%), or Atheist (6%). The vast majority, 70%, of patients, were diagnosed with advanced/metastatic cancers. Other medical diagnoses included severe/rare non-genetic conditions (11%), severe/rare genetic conditions (9%), blood dyscrasias (9%), and acquired immunodeficiency syndrome or AIDS (1%). Patients described their illness severity as extremely severe (43%), severe (36%), moderate (14%), and mild to not severe (7%). Further description of participant demographics and condition profiles are detailed in Table 1 and elsewhere [40].

The median (IQR) total NIH-HEALS score was 134 (120–145) (Table 1). The median (IQR) total CD-RISC-10 and MAAS scores in this cohort were 31 (25–35) and 67 (57–75), respectively. For each of these measures, higher scores represented better psychosocial-spiritual well-being, resilience, and mindfulness, respectively. NIH-HEALS, CD-RISC-10, and MAAS total scores did not differ between genders (p = 0.19, p = 0.88, and p = 0.97 respectively). Further analyses of the demographic characteristics of the present cohort are reported elsewhere [43].

**Fig. 1:**
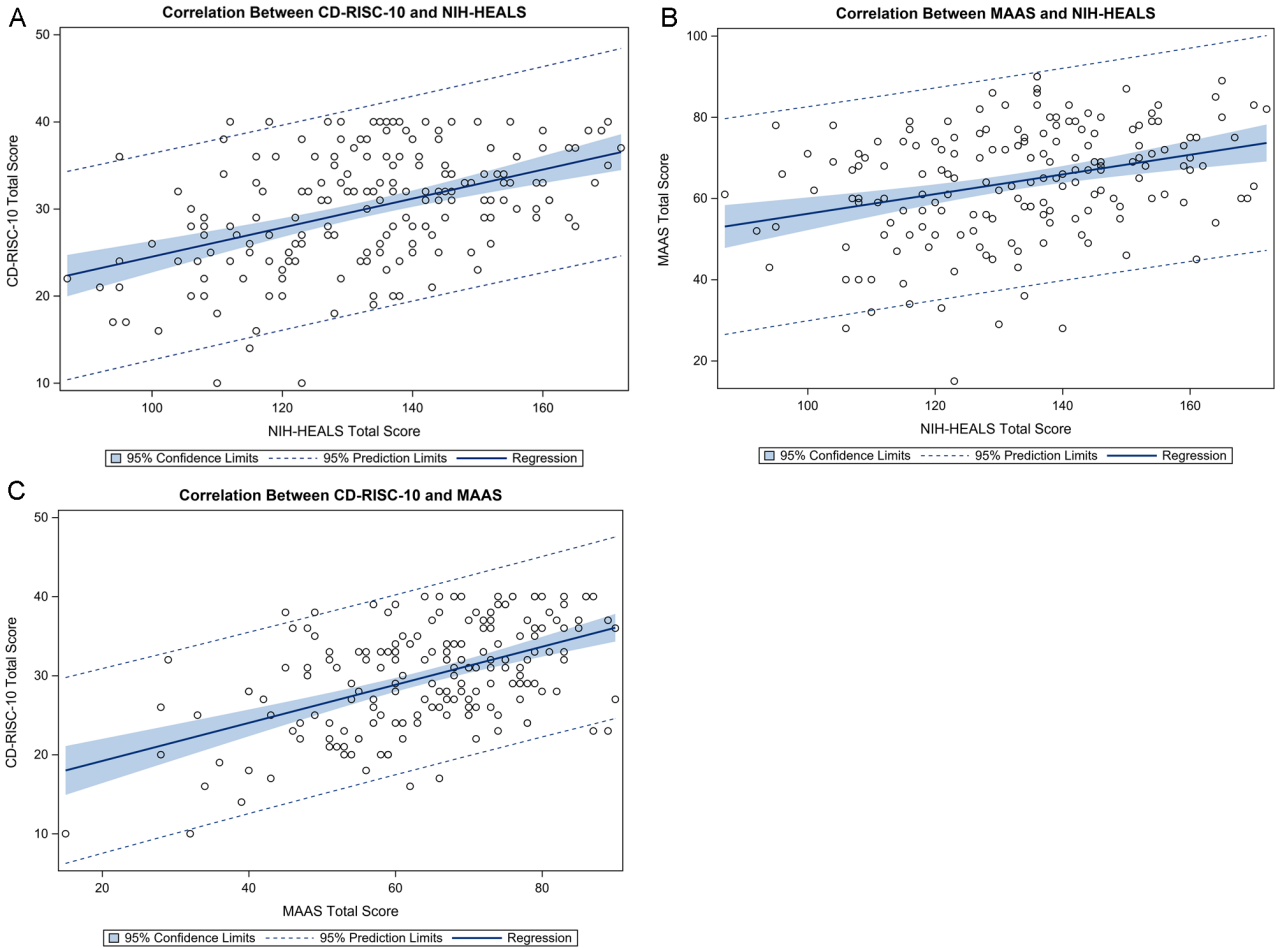
Correlation between NIH-HEALS, CD-RISC-10, and MAAS total scores in a cohort of patients with severe and/or life-limiting illnesses. Regression line and 95% confidence limits are shown. **A**) CD-RISC-10 and NIH-HEALS total scores were positively correlated (r_s_=0.44, p < 0.001, n = 178). **B**) MAAS and NIH-HEALS total scores were positively correlated (r_s_=0.32, p < 0.001, n = 179). **C**) CD-RISC-10 and MAAS total scores were positively correlated (r_s_=0.46, p < 0.001, n = 184)

The NIH-HEALS total score was positively correlated with CD-RISC-10 total score (r_s_=0.44, p < 0.001) and MAAS total score (r_s_=0.32, p < 0.001) (Fig. 1a, b). Similarly, NIH-HEALS factors, e.g., Connection, Reflection/Introspection, and Trust/Acceptance, were correlated with CD-RISC-10 and MAAS (Table 2). Further, CD-RISC-10 total score was positively related to MAAS total score (r_s_=0.46, p < 0.0001) (Fig. 1c).

**Table 2:** Correlations (r_S_) between CD-RISC-10, MAAS, and NIH-HEALS total scores and factors

Measure	MAAS Total Score	NIH- HEALS Total Score	NIH-HEALS Factors		
			Connection	Reflection & Introspection	Trust & Acceptance
CD-RISC-10 Total Score	0.46 p < 0.0001 (n = 184)	0.44 p < 0.001 (n = 178)	0.17 p = 0.022 (n = 186)	0.38 p < 0.001 (n = 184)	0.59 p < 0.001 (n = 185)
MAAS Total Score	--	0.32 p < 0.001 (n = 179)	0.16 p = 0.028 (n = 187)	0.21 p = 0.004 (n = 184)	0.41 p < 0.001 (n = 185)

Data are Spearman’s rho correlation coefficients (r_s_) with corresponding p-values and applicable n.

CD-RISC-10: Connor-Davidson Resilience Scale [41].

NIH-HEALS: NIH Healing Experiences of All Life Stressors [40].

MAAS: Mindful Attention Awareness Scale [42].

Additionally, NIH-HEALS, CD-RISC-10, and MAAS demonstrated an inverse correlation with current stress level. Patients reported their current level of stress as Extreme (3%), Severe (11%), Moderate (47%), Mild (29%), or No Stress (10%). Current level of stress was inversely related to NIH-HEALS (r_s_= -0.24, p = 0.001), CD-RISC-10 (r_s_=-0.31, p < 0.0001), and MAAS (r_s_=-0.37, p < 0.001).

## Discussion

The results of the current study indicate a relationship between psychosocial-spiritual well-being, resilience, and mindfulness in patients with severe or life limiting illness. Our results demonstrate that psychosocial-spiritual well-being is positively related to resilience (r_s_=0.44, p < 0.001), and there is a similar positive correlation between psychosocial-spiritual well-being and mindfulness (r_s_=0.32, p < 0.001). Additionally, resilience and mindfulness were positively correlated in this patient cohort (r_s_=0.46, p < 0.001). All three constructs were inversely related to current stress level.

Psychosocial-spiritual well-being captures the experience of healing in individuals facing major life stressors including a poor medical prognosis [40]. Patients with high levels of psychosocial-spiritual well-being report better coping skills and the ability to find meaning and hope in their experience [1]. The present study reports a positive relationship between resilience, mindfulness and each of the factors of psychosocial-spiritual well-being as measured by the NIH-HEALS. Resilience and mindfulness were related to the Connection factor which assesses the degree to which family, religion, and a religious community plays a role in one’s well-being. Both constructs were also related to Reflection & Introspection measuring the importance of finding meaning through attending to the present moment and appreciation for art and nature. Lastly, mindfulness and resilience were significantly correlated with the Trust & Acceptance factor which measures acceptance or feeling at peace with the diagnosis and trusting that caregivers, including family and friends, will respond to one’s needs for care.

Present findings illustrate a relationship between resilience and psychosocial-spiritual well-being that is consistent with the literature. Previous studies have established that resilience is associated with improvements in well-being including positive affect, quality of life and life satisfaction in patients [45]. Patients with higher levels of resilience cope better with their illness and report higher levels of optimism, positive affect, and self-efficacy [16, 46]. Similar evaluations conclude that resilience is also a protective factor against negative affect and psychological distress including anxiety and depression. A severe and life-limiting diagnosis can be traumatic for many patients. Effective and adaptive coping may rely, at least in part, on accepting one’s new state of health and well-being and maintaining a positive view of self. Resilient patients possess higher levels of self-efficacy that allows them to maintain a greater sense of control in life despite challenging circumstances [47]. This attitude may also influence clinical care and patient health as resilient patients are more likely to adhere and follow treatment instructions [48]. Psychological interventions targeting enhancement of resilience could, therefore, be promising to improve well-being and other health outcomes in patients facing serious illness.

Mindfulness is the practice of paying attention on purpose and nonjudgmentally to one’s experiences in the present moment. Mindfulness-based interventions have been shown to increase psychosocial-spiritual well-being in several clinical populations [49, 50]. In the present cohort, mindfulness was related to all three factors of NIH-HEALS including the Reflection & Introspection factor. Garland et al. (2017) suggest that mindfulness promotes well-being through a decentering and re-appraisal mechanism. During this process the individual reframes negative cognitions in a positive or neutral way which may enhance the perception of meaning in spite of adversity [51, 52]. Therefore, mindfulness may play a role in recovery and promote healing in patients by inviting an open-minded assessment of their diagnosis and encouraging to continue finding joy throughout the journey. Patients who are able to continue finding meaning in their situation are better equipped to cope with their diagnosis which contributes to greater satisfaction and quality of life. In other words, mindfulness can help patients find meaning and joy in their lives even when expectation for a cure might be unrealistic [53].

The present study also reports a positive correlation between mindfulness and resilience. The relationship between these constructs had previously been established among students and healthcare professionals including clinicians, nurses, and family members; however, few studies have investigated these correlations in patient groups [39]. Literature shows that resilience is an important construct that mediates the relationship between mindfulness and parameters of well-being including greater life satisfaction, higher positive affect, and lower negative affect [23]. We propose that a similar mechanism could also be at play in severely-ill patient populations. Mindfulness allows patients to view their situation in a positive light rather than ruminating on a poor prognosis. Resilient patients therefore find strength by redefining their sense of purpose and continue finding joy in relationships and activities [40].

Regarding clinical outcomes, present findings show that improvement in patient psychosocial-spiritual well-being, resilience, or mindfulness is related to reduced stress levels. This is consistent with literature as mindfulness-based interventions including the Mindfulness-Based Stress Reduction (MBSR) and the Mindfulness-Based Cognitive Therapy (MBCT) have been shown to reduce psychological stress and improve well-being in several clinical populations [54–56]. Similar improvements have also been reported when resilience is targeted [46, 57, 58]. Hence mindfulness interventions aimed at enhancing resilience are promising to lower negative affect and improve psychosocial-spiritual well-being in patients with serious illness.

The study limitations include reliance on self-rated measures and a correlational study design. More in-depth studies may be conducted to further characterize psychosocial-spiritual well-being, resilience, and mindfulness using clinical interviews or clinician-administered measures. Due to the correlational nature of the present study, the relationships found among study constructs do not establish cause and effect. Experimental designs are needed to further evaluate the associations among these and other related constructs in controlled settings. Nonetheless, the current findings suggest that interventions aimed at enhancing mindfulness and resilience may improve psychosocial-spiritual well-being and quality of life in patients with severe and life limiting illness. It should be noted, however, that generalization of results to other patient groups require additional investigations. Moreover, the NIH-HEALS is a newly validated scale. Future studies should investigate its various components in different cultures and subject groups. Additionally, the NIH-HEALS is a 35-item tool, a shorter version of this measure might be more appropriate for patients with severe illness and in other clinical populations.

## Data Availability

Information could be found on clinicaltrials.gov. Refer to NCT03871270. Note that the present study did not involve any clinical trial. More detail information can be shared upon request. Contact: Ninet Sinaii (sinaiin@cc.nih.gov) or Ann Berger (aberger@cc.nih.gov).
